# Supplementary material for: Multi‐dataset identification of innovative feature genes and molecular mechanisms in keratoconus
Source: J Cell Mol Med. 2024 Sep 19;28(18):e70079. doi: 10.1111/jcmm.70079 (PMC11412914; doi:10.1111/jcmm.70079)
Supplement: Supplementary file 2 — Material S2. [file JCMM-28-e70079-s002.zip › 7.GSEA analysis/5.RNF217-AS1/KEGG_GLYCOSAMINOGLYCAN_BIOSYNTHESIS_CHONDROITIN_SULFATE.html]

Details for gene set KEGG\_GLYCOSAMINOGLYCAN\_BIOSYNTHESIS\_CHONDROITIN\_SULFATE[GSEA]

|  || Dataset | RNF217-AS1.group.cls#h\_versus\_l |
| Phenotype | group.cls#h\_versus\_l |
| Upregulated in class | l |
| GeneSet | KEGG\_GLYCOSAMINOGLYCAN\_BIOSYNTHESIS\_CHONDROITIN\_SULFATE |
| Enrichment Score (ES) | -0.61350614 |
| Normalized Enrichment Score (NES) | -1.6293576 |
| Nominal p-value | 0.019607844 |
| FDR q-value | 0.3007285 |
| FWER p-Value | 0.55 |
Table: GSEA Results Summary

  

Fig 1: Enrichment plot: KEGG\_GLYCOSAMINOGLYCAN\_BIOSYNTHESIS\_CHONDROITIN\_SULFATE      
 Profile of the Running ES Score & Positions of GeneSet Members on the Rank Ordered List

  

| SYMBOL | TITLE | RANK IN GENE LIST | RANK METRIC SCORE | RUNNING ES | CORE ENRICHMENT || 1 | CHSY3 | na | 6045 | 0.182 | -0.1217 | No |
| 2 | CHPF | na | 15253 | 0.017 | -0.3822 | No |
| 3 | CHST11 | na | 15771 | 0.011 | -0.3940 | No |
| 4 | CHST13 | na | 16753 | 0.001 | -0.4219 | No |
| 5 | DSE | na | 17268 | -0.004 | -0.4355 | No |
| 6 | CHST7 | na | 17713 | -0.008 | -0.4459 | No |
| 7 | B3GAT3 | na | 20498 | -0.035 | -0.5161 | No |
| 8 | XYLT2 | na | 22464 | -0.056 | -0.5567 | No |
| 9 | CSGALNACT2 | na | 22643 | -0.058 | -0.5451 | No |
| 10 | CHSY1 | na | 22954 | -0.061 | -0.5364 | No |
| 11 | B4GALT7 | na | 25631 | -0.100 | -0.5846 | Yes |
| 12 | B3GAT2 | na | 26445 | -0.115 | -0.5748 | Yes |
| 13 | CHST12 | na | 26638 | -0.119 | -0.5460 | Yes |
| 14 | B3GALT6 | na | 27666 | -0.140 | -0.5352 | Yes |
| 15 | CHST15 | na | 27970 | -0.148 | -0.5013 | Yes |
| 16 | XYLT1 | na | 28083 | -0.151 | -0.4611 | Yes |
| 17 | CHPF2 | na | 28115 | -0.152 | -0.4183 | Yes |
| 18 | CHST3 | na | 31076 | -0.245 | -0.4329 | Yes |
| 19 | B3GAT1 | na | 31618 | -0.270 | -0.3708 | Yes |
| 20 | UST | na | 32165 | -0.301 | -0.2998 | Yes |
| 21 | CSGALNACT1 | na | 32753 | -0.342 | -0.2181 | Yes |
| 22 | CHST14 | na | 34698 | -0.954 | 0.0009 | Yes |
Table: GSEA details [plain text format]

  

Fig 2: KEGG\_GLYCOSAMINOGLYCAN\_BIOSYNTHESIS\_CHONDROITIN\_SULFATE      
 Blue-Pink O' Gram in the Space of the Analyzed GeneSet

  

Fig 3: KEGG\_GLYCOSAMINOGLYCAN\_BIOSYNTHESIS\_CHONDROITIN\_SULFATE: Random ES distribution      
 Gene set null distribution of ES for **KEGG\_GLYCOSAMINOGLYCAN\_BIOSYNTHESIS\_CHONDROITIN\_SULFATE**
